# Oral Medicines for Children in the European Paediatric Investigation Plans

**DOI:** 10.1371/journal.pone.0098348

**Published:** 2014-06-04

**Authors:** Diana A. van Riet – Nales, Erwin G. A. W. Römkens, Agnes Saint-Raymond, Piotr Kozarewicz, Alfred F. A. M. Schobben, Toine C. G. Egberts, Carin M. A. Rademaker

**Affiliations:** 1 Medicines Evaluation Board in the Netherlands (MEB), Department of Chemical Pharmaceutical Assessment, Utrecht, the Netherlands; 2 Utrecht University, Faculty of Science, Utrecht Institute for Pharmaceutical Sciences (UIPS), Department of Pharmacoepidemiology and Clinical Pharmacology, Utrecht, the Netherlands; 3 European Medicines Agency, Human Medicines Special Areas, London, United Kingdom; 4 European Medicines Agency, Quality of Medicines, London, United Kingdom; 5 University Medical Centre Utrecht, Department of Clinical Pharmacy, Utrecht, the Netherlands; Nottingham University, United Kingdom

## Abstract

**Introduction:**

Pharmaceutical industry is no longer allowed to develop new medicines for use in adults only, as the 2007 Paediatric Regulation requires children to be considered also. The plans for such paediatric development called Paediatric Investigation Plans (PIPs) are subject to agreement by the European Medicines Agency (EMA) and its Paediatric Committee (PDCO). The aim of this study was to evaluate the key characteristics of oral paediatric medicines in the PIPs and the changes implemented as a result of the EMA/PDCO review.

**Methods:**

All PIPs agreed by 31 December 2011 were identified through a proprietary EMA-database. PIPs were included if they contained an agreed proposal to develop an oral medicine for children 0 to 11 years. Information on the therapeutic area (EMA classification system); target age range (as defined by industry) and pharmaceutical characteristics (active substance, dosage form(s) as listed in the PIP, strength of each dosage form, excipients in each strength of each dosage form) was extracted from the EMA website or the EMA/PDCO assessment reports.

**Results:**

A hundred and fifty PIPs were included corresponding to 16 therapeutic areas and 220 oral dosage forms in 431 strengths/compositions. Eighty-two PIPs (37%) included tablets, 44 (20%) liquids and 35 (16%) dosage forms with a specific composition/strength that were stored as a solid but swallowed as a liquid e.g. dispersible tablets. The EMA/PDCO review resulted in an increase of 13 (207 to 220) oral paediatric dosage forms and 44 (387 to 431) dosage forms with a specific composition/strength. For many PIPs, the target age range was widened and the excipient composition and usability aspects modified.

**Conclusion:**

The EMA/PDCO review realized an increase in the number of requirements for the development of oral dosage forms and a larger increase in the number of dosage forms with a specific composition/strength, both targeting younger children. Changes to their pharmaceutical design were less profound.

## Introduction

On 26 January 2007, the Paediatric Regulation came into force with the aim to improve the information on medicines for children, to increase ethical drug research in paediatrics and to increase the availability of appropriately authorized medicines for the children of Europe [1]–[4]. The Regulation requires the submission of a Paediatric Investigation Plan (PIP) to the European Medicines Agency (EMA) for agreement by its Paediatric Committee (PDCO). The PIP defines the studies, measures and timelines necessary to ensure that data are collected supporting the authorization of the medicine in children and that such studies are safe to conduct. In addition, the PIP should include a description of the pharmaceutical development of the medicine proposed for future marketing [2], [3], [5]. The EMA/PDCO PIP decisions have a binding character and industry can only apply for marketing authorization of the (adult) medicine when the EMA has confirmed that the PIP was followed or a deferral was obtained [2], [6].

Estimation of the extent to which the Paediatric Regulation will meet one of its goals to enhance the availability of appropriately authorized paediatric medicines would necessitate an analysis of the trends observed over time in the availability of medicines for children of a particular age as authorized in each of the European member states. However, only few medicines with a PIP have reached marketing authorization already as the development of a new medicine may cost many years whereas the Regulation has only existed for a few [7], [8]. It is anticipated that a comparison of the paediatric medicines as originally proposed by industry in the PIPs and as finally agreed by the EMA/PDCO may provide a valuable prognostic estimate of the extent to which the Regulation will be able to achieve this goal.

Based on an analysis carried out on data covering one year (2009), the EMA's 5-year PIP evaluation report to the Commission stated that the EMA/PDCO raised many questions with respect to the pharmaceutical characteristics and the dosing of the medicines that were initially proposed by industry in the PIP [7], [9]. This study further expands on this analysis by an evaluation of the key characteristics of oral paediatric medicines in the PIPs and the changes implemented as a result of the EMA/PDCO review.

## Methods

### Study design

This retrospective study evaluated the characteristics of oral paediatric medicines in the PIPs. As the study did not contain human subjects, it was not subject to ethical approval according to the Dutch Medical Research Involving Human Subjects Act (WMO) [10]. The study protocol was approved by the EMA as part of the Memorandum of Understanding with the National Institute for Public Health and the Environment (RIVM). The data were retrieved from the EMA internet and supplemented with data from two proprietary EMA repositories. Protection of the proprietary data was assured by the RIVM and Medicines Evaluation Board (MEB) confidentiality rules for regulatory information. Only researchers that had signed an RIVM or MEB confidentiality agreement were allowed to extract and analyse the data. Data were anonymized after the data analysis.

### PIP selection

Original PIPs that successfully passed the EMA/PDCO review between 1 July 2007 and 31 December 2011 (agreed PIPs) were identified by a single researcher (ER) in a proprietary repository, the Paediatric Records Application database (PedRA). This database captures the main administrative, pre-clinical, clinical and (since October 2011) quality details of all PIPs submitted to and assessed by the EMA/PDCO. PIPs were included in this study if they contained an oral medicine for children between birth and 12 years of age (0 to 11 years old; 0–11 years) or a subset thereof. PIPs for oral vaccines and oral allergens were excluded. Oral medicines were defined as medicines that should be taken by mouth to be swallowed [11].

### Data extraction

A proposal for the development of a medicine for children in a specific age range does not assure that the medicine will be available in a dosage form(s) that is (are) sufficiently adapted to the age of the child from the minimum to the maximum of this age range, that the excipients in the dosage form(s) are safe for all the proposed ages, or that the proposed strength(s) allow the administration of all doses required [12], [13]. Therefore the following data were extracted by the same researcher for each of the included PIPs:

- administrative data: PIP-number, PIP-applicant, date of start of the procedure, date of final opinion/end of procedure;
- therapeutic area (EMA classification system) [3], [7];
- target age range (as defined by industry);
- pharmaceutical characteristics: active substance, dosage form(s) as listed in the PIP, strength(s) of (each of the) dosage form(s), excipients in each strength of a dosage form;
- aspects that are relevant to the practical use and/or acceptability of a paediatric medicine by health care professionals, caregivers or patients. Such aspects will be further referred to as usability aspects. Attention was put to the correct use of tablets and information was extracted on tablet size, tablet shape and the presence of break marks.

Data were extracted from the EMA website, its original source the proprietary PeDRA database or from the PIP assessment summary reports as downloaded from a second proprietary repository, the EMA Document Records and e-Archive Management database (DREAM). The extracted data comprised information from the PIP as submitted by industry at the start of the procedure (initial PIP) and as agreed with the EMA/PDCO at the end of the procedure (agreed PIP). All data were recorded and interpreted as outlined in Annex S1.

### Data Analysis

For each PIP, the target age range was categorized in the following groups: 0–5 months, 6–23 months, 2–5 years, 6–8 years, 9–11 years. In addition, data were categorized per type and subtype(s) of the dosage form(s) and the type(s) of preparation(s). A separate category further referred to as solid-liquid was created for dosage forms that were manufactured as a solid dosage form, but administered to the child as a liquid dosage form e.g. dispersible tablets. An oral preparation was defined as a subtype of an oral dosage form with a particular strength/concentration and with a particular excipient composition; e.g. a PIP containing film-coated tablets 50 mg and chewable tablets 5, 10 and 20 mg included one type of dosage form (tablets), two subtypes (film-coated and chewable tablets) and four preparations [13].

Descriptive analyses were conducted to evaluate the changes between the initial and agreed PIPs with respect to the number, therapeutic area, target age range and pharmaceutical characteristics of the oral, paediatric medicines. The analysis of the pharmaceutical aspects was conducted pair wise per PIP and group wise for all PIPs. A change in a dosage form subtype was defined as the addition, deletion or replacement of a subtype or as a proposal for a defined subtype in cases where this information was initially lacking e.g. age-appropriate formulation into oral suspension.

## Results

On 31 December 2011, the EMA/PDCO had agreed on 720 PIP applications and requests for a full waiver. A hundred fifty PIPs were included in this study (Annex S2, Annex S3).

### Therapeutic area

The agreed PIPs related to 165 indications in 16 of the 21 EMA therapeutic areas [3], [7]; 137 PIPs (91%) related to one, 10 PIPs (7%) to two and 3 PIPs (2%) to three areas. The main areas were infectious diseases (n = 28 PIPs, 19%), endocrinology/gynaecology/fertility/metabolism (n = 24 PIPs, 16%), cardiovascular diseases (n = 21 PIPs, 14%), oncology (n = 20 PIPs, 13%) and neurology (n = 13 PIPs, 9%). These areas were not changed as a result of the EMA/PDCO review.

### Target age range

**Figure 1:**
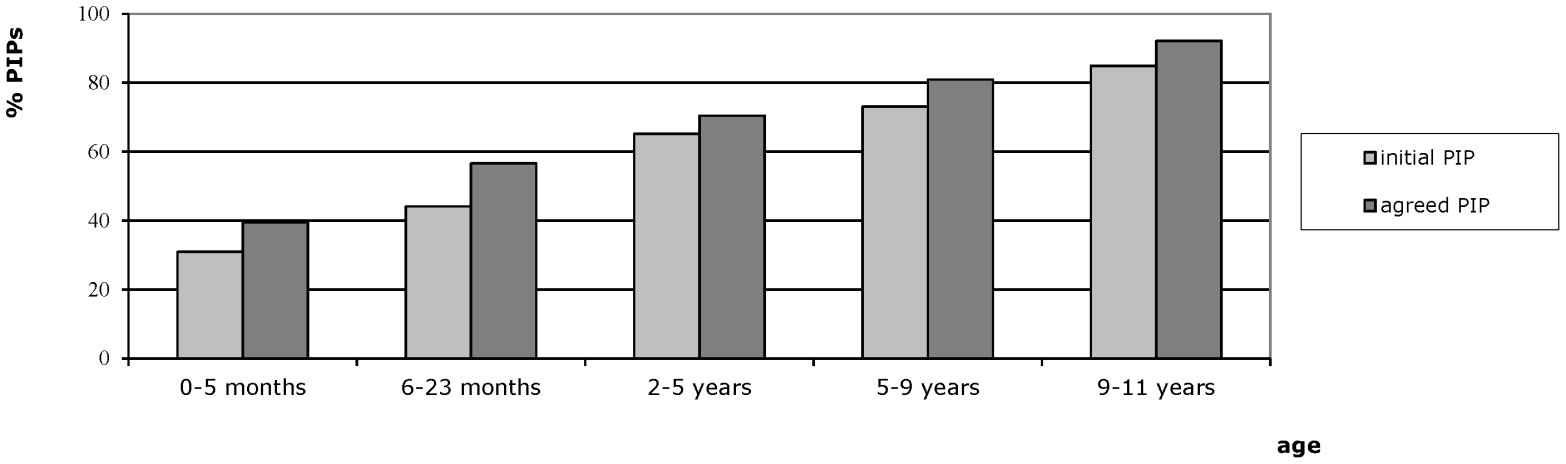
PIPs including at least one oral medicine for children 0–11 years.

The availability of authorized paediatric medicines on the European market largely varies with age with fewer medicines for younger children [14]. In order to promote the availability of authorized paediatric medicines in especially the youngest age groups, special attention is warranted to the target age range of medicines proposed for future marketing. Sixty of the agreed PIPs (40%) included at least one oral dosage form for children 0–5 months compared to 140 PIPs (93%) for children 9–11 years (Fig. 1).

As a result of the EMA/PDCO review, for 60 PIPs (40%) the lower age limit was extended to a younger age group, whereas for 6 PIPs (4%) it was set at an older age. For 2 PIPs (1%) the upper age limit was agreed at a younger age whereas for 3 PIPs (2%) it was set at an older age. Five PIPs (3%) were included for which initially a full waiver was requested i.e. these PIPs related to medicines for which industry had no initial intention to market a paediatric medicine. Nowadays, such waiver requests will be refused meaning industry has to submit a new PIP application.

### Pharmaceutical characteristics: pairwise comparison

Eighty-eight of the agreed PIPs (59%) included one, 54 PIPs (36%) two and 8 PIPs (5%) three or more types of an oral dosage form. Following the EMA/PDCO review, for 13 PIPs (9%) for which initially no oral dosage form was proposed, such a form was agreed; for 8 PIPs (5%) a dosage form other than initially proposed was agreed; for 8 (5%) PIPs one or several dosage forms were added and for 8 PIPs (5%) one or several dosage forms were deleted.

As a result of the EMA/PDCO review, for 60 PIPs (40%) changes were implemented with respect to the subtype, strength and/or excipient composition of the paediatric medicine. For 44 PIPs (29%) changes were implemented with respect to the subtype of the initially proposed dosage form. For 38 PIPs (25%) the medicine could be given in a wider range of strengths than initially proposed. Comparing the same subtypes of a dosage form in the initial and agreed PIP only, for 14 PIPs (9%) the number of strengths was increased and for 12 PIPs (8%) a strength was proposed whereas it was not before. Sixteen PIPs (11%) were changed with respect to the excipient composition.

### Pharmaceutical characteristics: group wise comparison

Information on the type(s) and subtype(s) of the dosage form(s) and preparations in the PIPs and the changes realized by the EMA/PDCO is provided in Table 1. Overall, the EMA/PDCO review led to a 6% increase in requirements for industry to market an oral paediatric dosage form and a more pronounced increase (11%) in requirements to market a specific oral preparation.

**Table 1:** Oral medicines in the Paediatric Investigation Plans (n = 150 PIPs); group wise comparison.

	oral dosage forms		oral preparations*	
	initial PIP n (%)	agreed PIP n(%)	initial PIP n (%)	agreed PIP n (%)
**All**	207 (100)	220 (100)	387 (100)	431 (100)
**tablets (all types)**	72	82	183	218
uncoated, immediate release	14	14	30	31
(film)-coated, immediate release	46	52	117	146
modified release, prolonged release or gastro-resistant	8	8	17	19
orodispersible / lyophilisate	3	3	7	7
chewable	6	6	11	14
**Capsules**	23	27	57	70
hard, immediate release	20	24	53	62
soft, immediate release	2	3	4	8
others	0	0	0	0
**powders/ granules**	9	13	10	16
**Liquids**	43	44	52	51
solution	25	24	29	27
suspension	14	11	16	12
unspecified liquids	4	9	7	11
**solid-liquids**	32	35	57	57
dispersible tablets	9	8	18	17
powder/granules for suspension	19	21	32	34
powder/granules for solution	6	6	6	6
**others / unspecified**	29	19	28	20

*a preparation is a subtype of a dosage form in a particular strength and with a particular excipient composition e.g. a PIP containing film-coated tablets 50 mg and chewable tablets 5, 10 and 20 mg represents one overall dosage form (tablets), two tablet subtype dosage forms (film-coated tablets and chewable tablets) and four preparations (film-coated tablets 50 mg, chewable tablets 5 mg, chewable tablets 10 mg, chewable tablets 20 mg).

**Figure 2:**
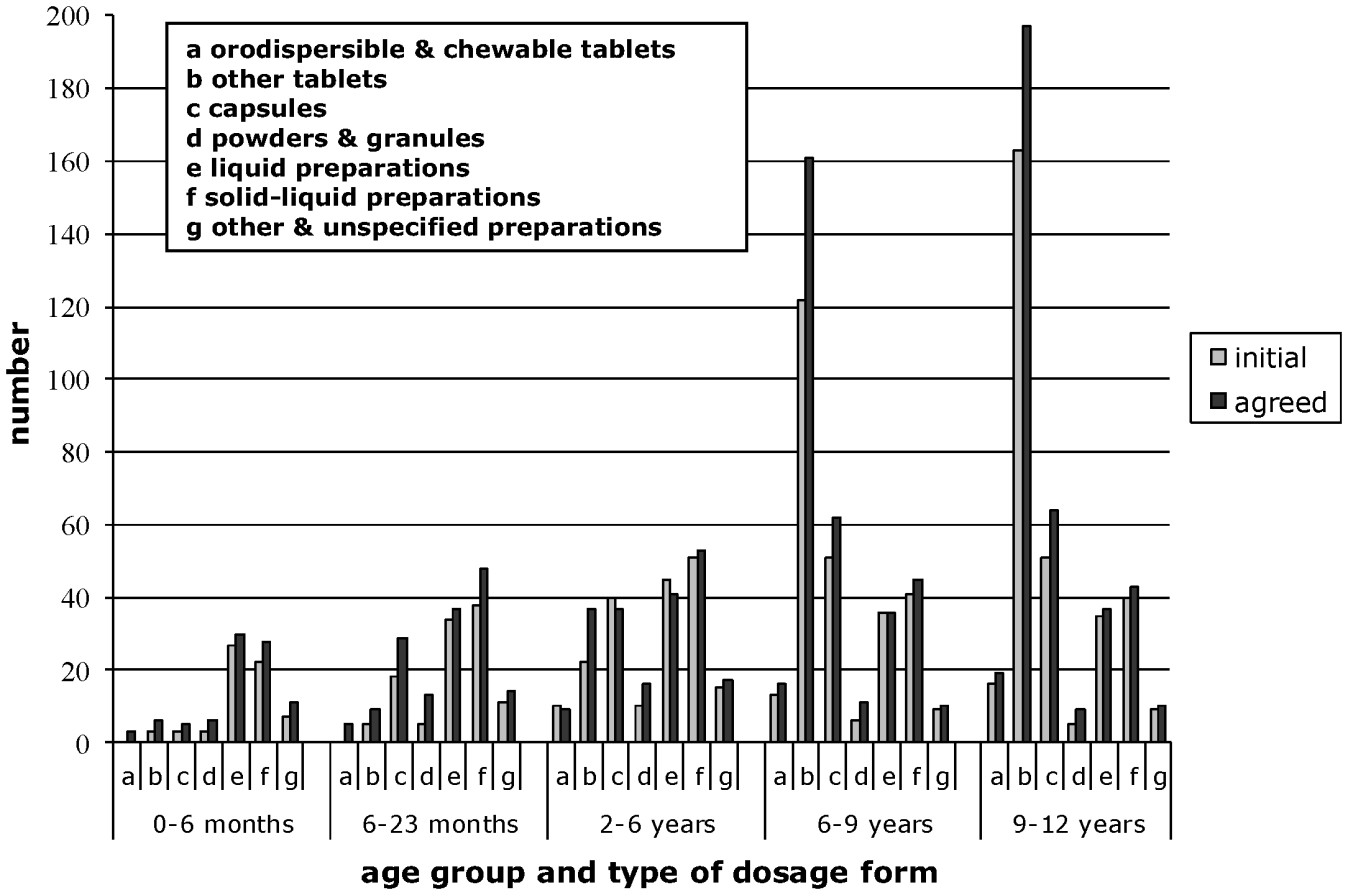
Oral preparations in the PIPs per target age group.

In the agreed PIPs, oral medicines for younger children were most commonly proposed as solid-liquid preparations (32% for children 0–5 months; 31% 6–23 months; 25% 2–5 years) and for older children as tablets (52% 6–8 years; 57% 9–11 years) (Fig. 2).

Detailed information on a selection of excipients with a potential cause for concern and their alternatives is provided in Table 2. Propylene glycol which may be relatively harmful for young children was included in three preparations agreed for children 0-23 months [15]–[17]. Only two of these preparations were proposed in the initial PIP. In all three preparations propylene glycol was included to dissolve the preservatives and/or active substance and the need for inclusion was debated with the pharmaceutical companies.

**Table 2:** Excipients in the Paediatric Investigation Plans (n = 150); group wise comparison.

		oral preparations* in the PIPs	
		initial PIP n (%)	agreed PIP n (%)
**all preparations**		387	431
*preparations with excipient information*		292 (100)	354 (100)
Solvents	propylene glycol	17	14
	ethanol	7	8
preservatives	methylparahydroxybenzoate	5	4
	methyl/propylparahydroxybenzoate%	9	11
	benzoates (E211)&	20	19
antioxidants	alpha-tocopherol	5	5
	butylhydroxyanisole (BHA)	2	5
	butylated hydroxytoluene (BHT)	3	2
	sodium phosphates	6	6
	potassium phosphates	2	2
colourants /opacifier	sunset yellow (E110)&	5	0
	tartrazine (E102)&	0	0
	carmoisine (E122)&	0	0
	ponceau 4r (E124)&	0	0
	quinoline yellow (E104)&	1	1
	allura red (E129)&	4	2
	iron oxide#	58	64
	opadry (any type)#	49	62
	titanium dioxide#	56	78
taste optimizers	sugars (incl. lactose)	107	117
	sugar alcohols	72	100
	sweeteners	27	44
	flavours	37	44

*a preparation is a subtype of a dosage form in a particular strength and with a particular excipient composition.

% excipient that has raised special attention by regulators in recent years due to a non-confirmed safety signal.

& studied by McCann et al. in the Southampton study and considered as potentially harmful [18]. The study conclusion was questioned by EFSA [19].

# may be used as a safe(r) alternative to the colourants studied by McCann et al. [18].

The colourants tartrazine (E102), quinolone yellow (E104), sunset yellow (E110), carmoisine (E122), ponceau 4R (E124) and allura red (E129) were investigated because of their allergic potential. It is noted that in 2007 these colourants were also associated with an increased risk on hyperactivity in children by McCann et all [18]. However, in 2008 the study was re-viewed and re-analysed by the European Food Safety Agency (EFSA). They concluded that the findings could not be used as a basis to change the acceptable daily intake of any of these colourants [19]. The colourants E110, E102, E122 and E124 were not included in any of the agreed PIPs; E104 was included in one preparation in one agreed PIP and E129 in two preparations in another PIP. Prior to the EMA/PDCO review these colourants were proposed in six preparations in three PIPs; E104 in an oral solution for children 2–11 years old, E110 in three film-coated tablets for children older than six years as well as in two oral solutions for children 2-11 years.

### Usability

For children 2–5 years, two agreed PIPs included each one small sized tablet (0–4 mm). Seven other PIPs included 17 medium sized tablets (5–9 mm) in a single strength/composition (4 uncoated, 6 film-coated, 5 modified release, 2 chewable in a single strength), whereas two of these seven PIPs also included 5 tablets in a single strength/composition sized 10 mm or larger (2 uncoated tablets, 2 modified release tablets and 1 chewable tablet). For six PIPs for which a tablet sized 5 mm or larger was agreed, there was no smaller tablet, oral liquid or other age-appropriate formulation required. Four of these PIPs were submitted in 2008; the remaining two in 2009 (Table 3).

**Table 3:** Tablet size and shape (n = 150 PIPs); group wise comparison children aged between 2 and 6 years.

	oral preparations in the PIPs					
	initial PIP n			agreed PIP n		
**all preparations**	193 (100)			210 (100)		
tablets* intended to be swallowed in their solid form	32			46		
immediate release	8			7		
film-coated	10			22		
modified release	4			8		
chewable	8			7		
oro-dispersible	2			2		
tablets* with information on size	11			24		
* small/medium/large*	*S*	*M*	*L*	*S*	*M*	*L*
immediate release	2	4	0	1	4	2
film-coated	1	1	0	1	6	0
modified release	0	3	0	0	5	2
chewable	0	0	0	0	2	1
oro-dispersible	0	0	0	0	0	0
tablets* with information on shape	16			25		
* round/oval/specified others*	*R*	*O*	*S*	*R*	*O*	*S*
immediate release	4	1	1	4	1	1
film-coated	2	0	1	3	5	0
modified release	3	0	0	5	2	0
chewable	3	1	0	3	1	0
oro-dispersible	0	0	0	0	0	0
tablets* with a break mark	3			9		
immediate release	1			2&		
film-coated	0			5		
modified release	0			0		
chewable	0			0		
oro-dispersible	2			2		

*tablets counted as the number of oral preparations i.e. differentiated to excipient composition and strength. A small tablet wa defined as 0–4 mm, medium sized 5–9 mm and large 10 mm or larger [21]. Oval tablets included those that were oblong or capsule shaped.

& related to the two tablets sized 10 mm or larger.

## Discussion

On 31 December 2011, the EMA/PDCO had agreed with 150 PIPs including an oral medicine for children 0–11 years. The EMA/PDCO review resulted in requirements for the future marketing of paediatric medicines in a wider age range than initially proposed by industry and with an increased number of oral dosage forms and strengths. The review also resulted in an increase in information on the medicines' excipients composition and usability aspects.

The Paediatric Regulation covers medicines administered through all routes of administration for children between birth and 18 years of age [2]. This study focused on oral medicines in order to allow an in-depth evaluation of their pharmaceutical characteristics. It was limited to children 0–11 years as older children can often be treated with the same oral medicines as adults. Although pharmaceutical characteristics of oral medicines for (pre-) term neonates require specific attention with respect to e.g. dosing volumes and compatibility with feeding tubes, all children 0–5 months were evaluated as a single group as essential dosing information was often not yet available in the PIP.

Changes in the target age range of a PIP as a result of the EMA/PDCO review often initiated a change in the (sub)types of the dosage forms and their characteristics. As the frequency of changes in the target age range hindered an age-specific evaluation of the achievements reached by the EMA/PDCO towards the pharmaceutical design of the proposed medicines, the pharmaceutical characteristics were evaluated per individual PIP as well as for all PIPs as a group.

The EMA's 5-year PIP evaluation report to the Commission indicated that industry insufficiently justified the choice of the excipients in relation to age, maximum daily dose and the possibility to replace potentially harmful excipients with those that are generally considered safer [7]. Our study showed that changes in the use of potentially harmful excipients were limited. This outcome does not contradict the above statement as the additional information that was requested by the EMA/PDCO may have justified the use of the proposed excipients on an overall positive benefit to risk evaluation of the medicine.

For three potentially harmful excipients their inclusion in oral medicines proposed for future marketing in children 0–11 years (PIPs) was compared to medicines currently authorized and commercially available for children between birth and 18 years of age (0–17 years) in the Netherlands (marketed products) [14]. Firstly, propylparahydroxybenzoate was related to a single safety signal. Its use i.e. proposed inclusion in liquid or solid-liquid preparations in the PIPs (10%) was comparable to its use in marketed products (11%). Secondly, propylene glycol may cause hyperosmolarity and lactic acidosis in young children [15]–[17]. Its use in oral medicines in the PIPs was less frequent (4%) than in marketed products (11%). Thirdly, the use of ethanol in the PIPs was less extensive (2%) than in marketed products (12%).

The EMA PIP evaluation report identified that patient acceptability should require better attention by industry. This aspect was not evaluated in this study because in the early PIPs, the need for acceptability studies was often discussed with the company during the assessment procedure, however if such studies were considered necessary this was not clearly stated in the list of binding terms of the PIP agreement. Instead in this study, a surrogate of patient acceptability was included by the analysis of excipients generally considered improving taste [20]. As a result of the EMA/PDCO review process, changes with respect to sugars, sugar alcohols, sweeteners and flavouring agents were generally uncommon.

In addition, a surrogate of child safety, patient usability and therewith patient acceptability was included by the analysis of tablet size. It is now increasingly accepted that small tablets may be applicable in young children [21]–[26]. However, the use of medium sized tablets is still discouraged, whereas the use of large sized tablets is generally considered unacceptable because of swallowing difficulties and the risk of choking [21], [27]. As a result of the EMA/PDCO review, companies had to provide more information on tablet size.

This study showed that tablets larger than 5 mm were agreed for children 2–5 years and tablets larger than 10 mm for children 6–11 years. Such tablets may be difficult to swallow by these age groups, unless they are taken as smaller parts [13], [21]. The majority of these “outsized” tablets were immediate release and film-coated tablets that may be broken, crushed or chewed, unless bio-availability or patient acceptability are affected [13]. Industry can justify the absence of changes to either of these aspects by several means including a scientific discussion or additional studies during paediatric development. Such studies were however not included in the list of binding terms of the PIP agreement.

The EMA also stressed that industry had to pay better attention to the practical aspects of administration, dosing accuracy and dosing flexibility [7]. Generally, smaller tablets may be easier to swallow and they may provide some dosing flexibility. However they may be more difficult to grip and hold by the patient hands. Tablets may also bear a break mark to ease swallowing or to adjust the dose [13]. Although commonly applied, the use of break marks has not been universally accepted. Firstly, the accuracy and ease of tablet breaking may have been demonstrated by companies, but not achieved by actual patients. This is because the accuracy and ease of breaking depend on hand function and the method of breaking [28]. Secondly, the use of tablet splitters is often inaccurate [29]. Tablets may also be broken, split or crushed and mixed with food or drinks to ease swallowing. However, these handlings may have an impact on the medicine's dosing accuracy, chemical stability and/or bio-availability [13]. All this favours the development of lower dosed tablets or alternative dosage forms. As a result of the EMA/PDCO review, changes in the number of liquid preparations, solid-liquid preparations or tablets with a break mark were generally uncommon.

Rather than discussing pharmaceutical issues on their own merit as has been done in this study, Sam et al. considered that the pharmaceutical development of paediatric medicines should be based on a multidisciplinary approach including safety, efficacy, manufacturability and patient access [30]. This opinion is consistent with the EMA/CHMP and EMA/PDCO overall benefit to risk approach for medicines entering the market [3], [31]. Thus, the oral preparations in the agreed PIPs may nevertheless contain some undesirable aspects that are either unavoidable (e.g. ethanol to dissolve the active substances) or open to further product optimization (e.g. taste).

This study has some limitations. Firstly, agreed PIPs may be modified on request of the pharmaceutical industry when information gained during the development of the paediatric medicine would make it necessary to revise the agreed plan. As a consequence, the target age range and pharmaceutical characteristics of the preparations in the agreed PIPs may vary to those actually proposed at the time of marketing authorization. In this study, the evaluation of PIP modifications was excluded because earlier PIPs would generally have undergone a higher number of modifications at 31 December 2011, hindering a fair comparison of all PIPs in the study period and putting overemphasis on earlier PIPs when industry and the EMA/PDCO were still learning [7].

Secondly, the summary report may not contain all details from the PIP that were relevant to this study. Moreover, the data in the summary report may have been interpreted slightly differently by the PDCO as intended by the EMA author or the pharmaceutical company, as the data were not fully reported. It was anticipated that the percentage error would be low.

Overall, this study confirms the likelihood that the children of Europe will gain better access to appropriately developed and authorized medicines. However, a group wise effect on the availability of medicines that are better tailored to children's needs could not (yet) be confirmed. In view of the ongoing learning process by industry and the EMA/PDCO, and an increase in the number of medicines licensed with a PIP, it is recommended to repeat this study in 5 to 10 years.

## Conclusions

The studies that were agreed by the EMA/PDCO to support the future marketing of a paediatric medicine were targeted at children of a younger age than those proposed by industry at the time of initial submission. For children 0–11 years, there was also an increase in the number of oral dosage forms and an even larger increase in the number of oral dosage forms with a particular strength or composition. The changes to the pharmaceutical characteristics of these dosage forms were less profound.

## Supporting Information

Annex S1 **Definitions and interpretation of information in the PIP.**
(DOC)

Annex S2 **PIP selection process.**
(DOC)

Annex S3 **PIPs included in the data analysis.**
(DOCX)
