# Supplementary material for: Oral Medicines for Children in the European Paediatric Investigation Plans
Source: PLoS One. 2014 Jun 4;9(6):e98348. doi: 10.1371/journal.pone.0098348 (PMC4045729; doi:10.1371/journal.pone.0098348)
Supplement: Annex S1 — Definitions and interpretation of information in the PIP. (DOC) [file pone.0098348.s001.doc]

**Annex S1: Definitions and interpretation of information in the PIP**

| **Target age group** | |
| --- | --- |
| **1** | When the selected target age group was specified in weight, then the corresponding age range was determined using the Dutch Denekamp scale [1]. If there was a difference between boys and girls, then the youngest age was reported. |
| **2** | If the term “pubertal and post-pubertal boys” was mentioned, then an age of 11 years was reported as the lower age limit [2]. |
| **3** | If the term “Tanner stage II” was mentioned, then a lower age limit of 9 years was reported for boys and 8 years for girls [3]. |
| **4** | If the term “preterm children” was mentioned, then an age range of 0 to 28 days was reported. |
| **5** | If the term “pre school children” was mentioned, then an age range of 2 to 6 years was reported. |
| **6** | If the term “school children” was mentioned, then an age range of 6 to 12 years was reported. |
| **7** | If the term “older children” was mentioned, then an age range of 9 to 12 years was reported. |
| **8** | If the term “adolescents” was mentioned, then an age range of 12 to 18 years was reported. |
| **9** | PIPs only indicated for girls after menarche were excluded (the mean age of menarche is above 12 years of age [4]). |
| **Tablets** | |
| **1** | Tablets were considered to be immediate release tablets, unless otherwise indicated. |
| **2** | Extended release, prolonged release and other modified release tablets may not be chewed or pulverized, unless otherwise indicated. |
| **3** | Small sized tablets (also referred to as mini-tablets) were considered to be smaller than 5 mm, unless otherwise indicated [5,6]. |
| **4** | Medium sized tablets were considered to be 5 to 10 mm, unless otherwise indicated [5]. |
| **5** | Large sized tablets were considered to be 10 to 15 mm, unless otherwise indicated [5]. |
| **6** | Very large sized tablets were considered to be larger than 15 mm, unless otherwise indicated [5]. |
| **7** | If the tablet size was not provided in the PIP, then it was considered that the tablets were large. |
| **8** | Coated tablets were considered to be neutral in taste, unless otherwise indicated. |
| **Capsules** | |
| **1** | Capsules were considered to be immediate release hard gelatin capsules, unless otherwise indicated. |
| **2** | Immediate release capsules were considered to be suitable for opening, unless otherwise indicated. |
| **3** | Modified release capsules were not considered to be suitable for opening, unless otherwise indicated. |
| **4** | Capsules were considered to be neutral in taste, unless otherwise indicated. |
| **5** | Small sized capsules were considered to be smaller than capsule size 3, unless otherwise indicated. |
| **6** | Medium sized capsules were considered to be size 2 or 3, unless otherwise indicated. |
| **7** | Large sized capsules were considered to be capsules size 0 and 1, unless otherwise indicated |
| **8** | Very large sized capsules were considered to be larger than size 0, unless otherwise indicated. |
| **9** | If the capsule size was not provided in the PIP, then the capsules were considered to be large. |
| **Multiparticulate formulations** | |
| **1** | Pellets were considered as granules. |
| **Dosage forms** | |
| **1** | Dosage forms which were proposed for children 12-18 years of age only were excluded. |
| **2** | If the applicant proposed several possible dosage forms from which eventually one would be chosen for marketing, then all proposed dosage forms were considered. |
| **Dosage forms and age** | |
| **1** | Tablets smaller than 5 mm were considered to be for children from 2 years of age, unless otherwise indicated [5,6]. |
| **2** | Tablets from 5 to 10 mm were considered to be for children from 6 years of age, unless otherwise indicated [5,6] |
| **3** | Tablets from 10 up to 15 mm were considered to be for children from 12 years of age, unless otherwise indicated[5]. |
| **4** | Tablets from 15 mm were considered to be for adults from 18 years of age only, unless otherwise indicated [5]. |
| **5** | Chewable tablets were considered to be for children from 2 years, unless otherwise indicated [7]. |
| **6** | Orodispersible tablets were considered to be for children from 1 month, unless otherwise indicated [7]. |
| **7** | Capsules smaller than size 3 that must be swallowed intact are considered to be for children from 6 years of age. |
| **8** | Capsules size 2 and 3 that must be swallowed intact are considered to be for children from 9 years of age. |
| **9** | Capsules size 0 and 1 that must be swallowed intact are considered to be for children from 12 years of age. |
| **10** | Capsules that may be opened prior to use in order to give their contents (powder, granules, liquid) as such are considered to be for children from 6 months. |
| **11** | Liquid preparations (solutions, suspensions, emulsions, drops) are considered to be for children from birth, unless otherwise indicated [8]. |
| **12** | Powders, granules, pellets for solution, dispersion or suspension are considered to be for children from birth, unless otherwise indicated [8]. |
| **13** | Powder, granules and pellets that are smaller than 2 mm and that are to be administered in their solid form are considered to be for children from 6 months, unless otherwise indicated [8]. |
| **Excipients** | |
| **1** | Trade marked coloring agents and flavorings were considered as a single excipient. |
| **2** | Printing ink was not considered as an excipient in this study. |
| **3** | The excipient composition of the capsule shell was not considered. |
| **4** | If the composition of a preparation was not provided in the summary report but it was clear from the summary report that the pharmaceutical information was identical to that of a licensed product, then the data were extracted from the product’s SmPC at the EMA or MEB website. |
| **8** | If the applicant provided several possible options for one type of excipient (e.g. preservative, sweetening agent) of which one ultimately would be selected for the marketed product, then all proposed excipients were considered. |
| **Strength** | |
| **1** | If a range of doses were proposed but no specific doses were mentioned, then the nature and pharmaceutical characteristics were only reported for the lower and upper limit of the range. |
| **2** | If no strength was provided, then the nature and pharmaceutical characteristics were considered as applicable to one strength only. |
| **3** | If the applicant proposed several strengths for one subtype of a dosage form and only provided the composition for some of these strengths, then the assumption was made that the same composition would apply to the strengths for which information was missing, unless it was indicated that a change in composition would be considered. |
| **Request on a full waiver** | |
| **1** | If the applicant requested a full waiver and the PDCO refused this request, then no data were reported at day 0, unless the PIP was supplemented with the requested data. |

**References**

1. Nederlands Kenniscentrum voor Farmacotherapie bij Kinderen. Denekamp schaal. Nederlands Kinderformularium. Available: <http://www.kinderformularium.nl/search/index.php?content=denekamp>. Accessed 1 September 2013.

2. Sizonenko PC. (1987) Normal sexual maturation. Paediatrician 14(4):191-201.

3. Berberoglu M. (2009). Precocious puberby and normal variant puberty: definition, etiology, diagnosis and current management. J Clin Res Endocrinol 1(4):164-174.

4. Parent AS, Tellmann G, Juul A, Skakkebaek NE, Toppari J et al. (2003) The timing of normal puberty and the age limits of sexual precocity: variations around the world, secular trends and changes after migration. Endocr Rev 24(5):668-693.

5. European Medicines Agency Committee for Medicinal Products for Human use. Draft guideline on the development of medicines for paediatric use. (2011) (EMA/CHMP/QWP/180157/2011). Available: <http://www.ema.europa.eu/docs/en_GB/document_library/Scientific_guideline/2011/06/WC500107908.pdf>. Accessed 1 September 2013.

6. Van Riet-Nales DA, de Neef BJ, Schobben AFAM, Ferreira JA, Egberts TCG et al. (2013) Acceptability of different oral formulations in infants and preschool children. Arch Dis Child. 98(9):725-31.

7. European Medicines Agency Committee for Medicinal Products for Human use. (2006) Reflection paper on formulations of choice for the paediatric population (EMEA/CHMP/PEG/194810/2005). Available: <http://www.ema.europa.eu/docs/en_GB/document_library/Scientific_guideline/2009/09/WC500003782.pdf>. Accessed 1 September 2013.

8. European Medicines Agency Committee for Medicinal Products for Human Use and the European Medicines Agency Paediatric Committee. (2013) Guideline on the development of medicines for paediatric use (EMA/CHMP/QWP/805880/2012 Rev. 2). Available: <http://www.ema.europa.eu/docs/en_GB/document_library/Scientific_guideline/2013/07/WC500147002.pdf>. Accessed 1 September 2013.
