# Supplementary material for: Oral Medicines for Children in the European Paediatric Investigation Plans
Source: PLoS One. 2014 Jun 4;9(6):e98348. doi: 10.1371/journal.pone.0098348 (PMC4045729; doi:10.1371/journal.pone.0098348)
Supplement: Annex S2 — PIP selection process. (DOC) [file pone.0098348.s002.doc]

**Annex S2: PIP selection process**

PIPs submitted to the EMA

up to 31 December 2011

n=1241

PIPs with a published

opinion or decision

n=720

full waiver on PIP granted

n=219

PIPs refused

n=4

PIPs with same active substance

& indications as other PIP

n=15

*excluded*

*excluded*

*excluded*

agreed PIPs

with a proposal for a paediatric trial

n=482

PIP including preparation(s)

for non oral use only

n=270

*excluded*

PIP relating to an

oral vaccine

n=1

*excluded*

PIP relating to an

oral allergen

n=52

*excluded*

PIP including preparation(s) for oral use in children > 12 years only

n=9

*excluded*

agreed PIP including at least one oral preparation for children 0-12 years

n=150
