# Supplementary material for: Oral Medicines for Children in the European Paediatric Investigation Plans
Source: PLoS One. 2014 Jun 4;9(6):e98348. doi: 10.1371/journal.pone.0098348 (PMC4045729; doi:10.1371/journal.pone.0098348)
Supplement: Annex S3 — PIPs included in the data analysis. (DOCX) [file pone.0098348.s003.docx]

**Annex S3: PIPs included in the data analysis**

EMEA-000049-PIP01-07

EMEA-000073-PIP01-07

EMEA-000065-PIP01-07

EMEA-000070-PIP01-07

EMEA-000122-PIP01-07

EMEA-000116-PIP01-07

EMEA-000132-PIP01-07

EMEA-000115-PIP01-07

EMEA-000114-PIP01-07

EMEA-000154-PIP01-07

EMEA-000144-PIP01-07

EMEA-000052-PIP01-07

EMEA-000041-PIP01-07

EMEA-000078-PIP01-07

EMEA-000170-PIP01-07

EMEA-000183-PIP01-08

EMEA-000191-PIP01-08

EMEA-000196-PIP01-08

EMEA-000200-PIP01-08

EMEA-000153-PIP01-07

EMEA-000019-PIP02-07

EMEA-000018-PIP01-07

EMEA-000038-PIP01-07

EMEA-000093-PIP01-07

EMEA-000087-PIP01-07

EMEA-000221-PIP01-08

EMEA-000222-PIP01-08

EMEA-000081-PIP01-07

EMEA-000022-PIP01-07

EMEA-000237-PIP01-08

EMEA-000245-PIP01-08

EMEA-000062-PIP01-07

EMEA-000054-PIP01-07

EMEA-000274-PIP01-08

EMEA-000055-PIP01-07

EMEA-000279-PIP01-08

EMEA-000283-PIP01-08

EMEA-000288-PIP01-08

EMEA-000290-PIP01-08

EMEA-000317-PIP01-08

EMEA-000300-PIP01-08

EMEA-000008-PIP01-07

EMEA-000325-PIP01-08

EMEA-000331-PIP01-08

EMEA-000332-PIP01-08

EMEA- 000012-PIP01-07

EMEA-000353-PIP01-08

EMEA-000005-PIP01-07

EMEA-000362-PIP01-08

EMEA-000365-PIP01-08

EMEA-000389-PIP01-08

EMEA-000391-PIP01-08

EMEA-000020-PIP01-07

EMEA-000409-PIP01-08

EMEA-000430-PIP01-08

EMEA-000434-PIP01-08

EMEA-000458-PIP01-08

EMEA-000459-PIP01-08

EMEA-000463-PIP01-08

EMEA-000470-PIP01-08

EMEA-000467-PIP01-08

EMEA-000477-PIP01-08

EMEA-000478-PIP01-08

EMEA-000487-PIP01-08

EMEA-000485-PIP01-08

EMEA-000491-PIP01-08

EMEA-000480-PIP01-08

EMEA-000496-PIP01-08

EMEA-000511-PIP01-08

EMEA-000533-PIP01-08

EMEA-000543-PIP01-09

EMEA-000551-PIP01-09

EMEA-000553-PIP01-09

EMEA-000567-PIP01-09

EMEA-000573-PIP01-09

EMEA-000576-PIP01-09

EMEA-000582-PIP01-09

EMEA-000583-PIP01-09

EMEA-000601-PIP01-09

EMEA-000617-PIP01-09

EMEA-000627-PIP01-09

EMEA-000651-PIP01-09

EMEA-000019-PIP06-09

EMEA-000694-PIP01-09

EMEA-000709-PIP01-09

EMEA-000718-PIP01-09

EMEA-000720-PIP01-09

EMEA-000727-PIP01-09

EMEA-000734-PIP01-09

EMEA-000745-PIP01-09

EMEA-000339-PIP02-09

EMEA-000774-PIP01-09

EMEA-000777-PIP01-09

EMEA-000780-PIP01-09

EMEA-000804-PIP01-09

EMEA-000822-PIP01-09

EMEA-000828-PIP01-09

EMEA-000235-PIP02-10

EMEA-000463-PIP02-10

EMEA-000637-PIP02-10

EMEA-000912-PIP01-10

EMEA-000927-PIP01-10

EMEA-000972-PIP01-10

EMEA-000425-PIP02-10

EMEA-000084-PIP02-10

EMEA-000597-PIP02-10

EMEA-001030-PIP01-10

EMEA-001034-PIP01-10

EMEA-000007-PIP01-07

EMEA-000788-PIP02-11

EMEA-000100-PIP01-07

EMEA-000063-PIP01-07

EMEA-000498-PIP01-08

EMEA-000625-PIP01-09

EMEA-000636-PIP01-09

EMEA-000716-PIP01-09

EMEA-001003-PIP01-10

EMEA-001057-PIP01-10

EMEA-000455-PIP02-10

EMEA-001098-PIP01-10

EMEA-001103-PIP01-10

EMEA-000499-PIP02-10

EMEA-000832-PIP01-10

EMEA-000170-PIP02-10

EMEA-000969-PIP01-10

EMEA-000696-PIP02-10

EMEA-001005-PIP01-10

EMEA-000726-PIP01-09

EMEA-000671-PIP01-09

EMEA-001094-PIP01-10

EMEA-000580-PIP01-09

EMEA-000115-PIP02-09

EMEA-000970-PIP01-10

EMEA-000452-PIP02-10

EMEA-000816-PIP02-10

EMEA-000360-PIP01-08

EMEA-000093-PIP02-10

EMEA-001078-PIP01-10

EMEA-000982-PIP01-10

EMEA-000997-PIP01-10

EMEA-001113-PIP01-10

EMEA-001061-PIP01-10

EMEA-000440-PIP01-08

EMEA-000637-PIP01-09

EMEA-000816-PIP01-09

EMEA-000342-PIP01-08

EMEA-000315-PIP01-08

EMEA-000335-PIP01-08

EMEA-000347-PIP01-08

EMEA-000350-PIP01-08
